# How Taiwan prevented the outbreak of COVID-19: a focus on psychological strategies and measures

**DOI:** 10.1017/S1041610220001568

**Published:** 2020-07-22

**Authors:** Tzung-Jeng Hwang, Yi-Ting Lin, Lih-Jong Shen, Liyu Tang, Maria I. Lapid

**Affiliations:** 1 Department of Psychiatry, College of Medicine and National Taiwan University Hospital, National Taiwan University, Taipei, Taiwan, R.O.C.; 2 Department of Mental and Oral Health, Ministry of Health and Welfare, Taipei, Taiwan, R.O.C.; 3 Taiwan Alzheimer’s Disease Association, Taipei, Taiwan, R.O.C.; 4 Department of Psychiatry and Psychology, Mayo Clinic, Rochester, MN, USA

The COVID-19 outbreak is unanticipated and unprecedented. As of June 6, 2020, the global number of confirmed cases is more than 6.6 million, with almost 400 thousand deaths (Dong *et al.*, 2020). Though Taiwan and Mainland China are geographically close and frequent contacts occur between the people, Taiwan only has 440 cases (the majority of which are imported) and 7 deaths (4 aged above 60 years). In particular, no local cases have been identified since April 13 (Taiwan Centers for Disease Control, 2020). In comparison, China has 84,181 confirmed cases and 4,638 deaths to date (Dong *et al.*, 2020). The success of Taiwan in controlling the spread of COVID-19 is due to critical, timely, and coordinated general and psychological preventive strategies and measures. In the commentary, we describe these crucial experiences and successful interventions relevant in the geriatric population, particularly in people with dementia (PWD).

The Taiwan government established the National Health Command Center (NHCC) one year after the severe acute respiratory distress syndrome (SARS) outbreak in 2003. The NHCC acts as the operational command point for direct communications among central, regional, and local authorities. On December 31, 2019, the Taiwan Centers for Disease Control (CDC) implemented inspection measures for inbound flights from Wuhan, China, in response to reports of an unidentified outbreak (Taiwan Ministry of Health and Welfare, 2020). The Central Epidemic Command Center (CECC), a part of NHCC, was officially activated by the CDC on January 20, 2020, when the first case of the coronavirus arrived from Wuhan to Taiwan. In the following 5 weeks, the CECC rapidly generated and applied at least 124 action items including border control from the air and sea, case identification, quarantine of suspicious cases, proactive case finding, resource allocation, public education and reassurance, formulation of policies toward schools and childcare, and financial relief to businesses (Chiu *et al.*, 2020)(Wang *et al.*, 2020). In addition, the government started to implement a series of specific psychological preventive strategies and measures for several target populations.

## Psychological preventive strategies and measures

### For the general population

Daily COVID-19 briefings were held with the Minister of Health and Welfare on the latest information on local epidemic and government policies (Taiwan Ministry of Health and Welfare, 2020). These briefings effectively reduced feelings of uncertainty, anxiety, and panic. Additionally, advice on coping strategies in the face of the pandemic stress was provided on official websites and through social media (Taiwan Centers for Disease Control, 2020). For individuals, a national hotline was established and made accessible for the general population in January 2020. There were between 248 and 337 calls per month during this period. As for the calls, the most common reason was related to questions about quarantine/isolation measures (28%), followed by psychological problems/concerns (24%) and work/financial problems (15%). Later, counseling hotlines were set up by individual local governments to provide telephone consultation.

### For the vulnerable populations

For the elderly, children, people with chronic medical diseases or mental disorders, and those financially disadvantaged, the Taiwan government had special recommendations to enhance the mental health of these vulnerable populations (Department of Mental and Oral Health of Taiwan Ministry of Health and Welfare, 2020). Official letters were sent to encourage relevant professional organizations to pay special attention to and to take actions to protect them from being adversely impacted by the pandemic. For example, the Taiwanese Society of Geriatric Psychiatry (TSGP) and Taiwan Alzheimer’s Disease Association (TADA) provided a counseling hotline to lend necessary support to psychogeriatric patients and their carers. The two organizations also had press releases with relevant information and practical tips for patients and caregivers.

### For those who were home quarantined or isolated

A total of around 130,000 people have been quarantined or isolated until late April. Starting in February, the NHCC set up a Call Center team composed of psychologists and later nurse volunteers who proactively reached out to offer counseling to people who were quarantined at home or isolated. A very low proportion of subjects (about 0.3%) expressed any need for counseling. Among them, the main problem was emotional disturbance (40%), including anxiety, dysphoria, anger, and sleep problems, followed by occupational/life adaptation problems (17%). From March 1, the local governments also set up hotlines to provide further psychological counseling for the same group of people. One small sample survey (N = 40) from Kaohsiung City showed that the chief complaint was emotional disturbance (accounting for more than half of the subjects), similar to NHCC data, as mentioned above.

### For those who were compulsorily quarantined

There were a few people (N = 32) who could not follow the regulations of self-quarantine (e.g. went out shopping or playing during self-quarantine period) and were therefore sent to stay in a government-designated place for compulsory quarantine. When the psychological needs of these subjects were detected, they were offered access to counseling with government-deployed psychologists from 7 core psychiatric institutes via a dedicated phone line. Among them, 18 (56%) had emotional disturbance and 15 (49%) had questions regarding inconvenience from quarantine, but only 12 (37.5%) required psychological counseling.

### For hospitalized subjects with COVID-19

The psychiatry and clinical psychology consultation-liaison team of each hospital provided consultations for people admitted due to confirmed or suspected COVID-19 infection.

### For the bereaved family

Seven patients died from COVID-19. When family members needed grief management, local governments arranged psychological counseling and bereavement support through the community mental health centers, though only a few were available.

### For frontliners

Frontliners include epidemic prevention health workers, physicians, nurses, and all related personnel. The Taiwan government advised the administrative department of each hospital to perform internal surveillance and provide proper psychological interventions when necessary. Articles and papers regarding psychological crisis intervention for epidemic prevention professionals were made available on the official website (Department of Mental and Oral Health of Taiwan Ministry of Health and Welfare, 2020).

### For PWD and their carers

At the national level, the government implemented rules for controlling visitors to long-term care facilities. In one facility where a resident was confirmed to be infected with COVID-19, all the other residents and staff were tested. The facility residents were evacuated to hospitals and other facilities for quarantine. Fortunately, only one nurse staff was infected, and no PWD was infected.

The TADA raised awareness of the needs of PWD in epidemic prevention through a press release in February 2020 and helped the public to understand the difficulties of PWD to follow the safety measures like putting on masks, etc. TADA developed the Reference Handbook of Dementia Care Responding to COVID-19 in March 2020 and produced videos of interactive exercise (Taiwan Alzheimer Disease Association, 2020). The handbook and videos were shared via social media or press releases. Health Promotion Administration of the Ministry of Health and Welfare forwarded the handbook to all local governments and shared these videos on the official website. The English version of the handbook and these videos were also posted on the official Alzheimer’s Disease International (ADI) website to share with people around the world (Alzheimer’s Disease International, 2020).

To help make daily life in quarantine bearable, TADA collected familiar old TV programs and shared via social media to help PWD and their caregivers to stay at home with better choices to arrange regular activities. When many usual programs were shut down during this period, TADA organized on-line events for PWD and caregivers. It also shared creative ways and strategies to help PWD wear facial masks (Taiwan Alzheimer Disease Association, 2020). Because violation of the epidemic prevention regulation (such as wearing a mask) would incur penalties, TADA advocated for the PWD, and now the latter are not liable for a violation if they show the certification of disability.

## Other critical factors for effective prevention: culture and regulations

In addition to those mentioned above, culture also plays a crucial role in effective prevention. The majority of Taiwanese people were willing to cooperate with relevant regulations, including mask-wearing, which was critical in preventing viral transmission (Chu *et al.*, 2020). In contrast, many citizens in European countries and the United States refused to wear a mask. A recent survey of 1098 Taiwanese citizens above age 20 showed 97.5% believed that being infected with COVID-19 was severe, and more than 90% could answer knowledge related to COVID-19 (such as preventive method, infection route, vaccine, etc.) correctly. Another survey of 2132 Taiwanese citizens revealed there was sufficient knowledge of and consensus on government’s epidemic preventive measures between individuals and families, and most people showed high levels of cooperation with regulations and voluntary mask-wearing (Hsu *et al.*, 2020). One interesting study found increased Google searches for “wash hands” were correlated with a slower spread of COVID-19 across countries (Lin *et al.*, 2020). Taiwan is one of the countries with the largest “google trend” values for this search, suggesting not only citizens’ awareness of enhancing hand hygiene but also implies a certain level of self-discipline.

In addition to citizens’ cooperation and self-discipline, the Taiwanese government also imposed many crucial regulations on the prevention and control of COVID-19. For example, strict regulations on home isolation/quarantine of COVID-19 confirmed cases imposed fines up to NT$1,000,000 for noncompliance under the Special Act for Prevention, Relief, and Revitalization Measures for Severe Pneumonia with Novel Pathogens. Early implementation of regulations on face mask distribution and wearing are other critical factors for success. The Taiwanese government ensured adequate stockpiles of surgical and N95 masks even before the first case was reported. Subsequently, the government implemented a series of regulations and measures to ensure universal access of face mask to all citizens, such as an initial ban on exportation, price-fixing of masks to prevent bidding wars, and rationing of surgical masks to prevent panic buying (Su *et al.*, 2020).

## Outcomes and challenges

The overall outcomes thus far are excellent. A survey of 1098 people above age 20 in late April showed that as high as 92.8% were confident regarding what the government has done in response to the COVID-19 pandemic. The results of emotional disturbance screening using the Brief Symptom Rating Scale (Lee *et al.*, 2003) showed 22% feeling nervous, 16% depressed, 14% easily distressed/irritated, and 15% having a sleep disturbance, but most of these symptoms were mild. In terms of overall severity of emotional disturbance, 94.7% were clinically nonsignificant (total scores 0–5), with only 5.3% reached a clinically significant level (total score equal or greater than 5), suggesting people, in general, were not psychologically distressed.

Despite these good outcomes, there are still challenges ahead. First, because of the low rates of infection, it remains to be seen whether the system will continue to work effectively when faced with a massive outbreak. Second, there are more and more foreigners living in Taiwan who may not be able to access timely help (especially psychological assistance) because of the language problem. Third, for the vulnerable population (such as older adults and PWD), there may be a need for more government-initiated actions, as many of the psychological interventions were initiated by non-government organizations. Part of the reason is inadequate budget for mental health at the national level. Fourth, because the majority of the population were not infected and with no immunity to the coronavirus, there is always a risk of another significant outbreak unless an effective vaccine becomes available.

## Conclusion

Taiwan has successfully prevented the outbreak of COVID-19 by taking a series of effective measures to control the spread of coronavirus. There are several important factors: (1) learning from the past SARS experiences, (2) having a well-functioning national healthcare system and NHCC in preparation for any major outbreak, (3) responding quickly and effectively, with advanced technology and consistent actions from central to local government, (4) transparency in communications and policies (including resource allocation), (5) efficient general and psychological strategies and measures, including regulations on face mask-wearing and punishment of those who break the rule of home isolation/quarantine. There is room for improvement in the mental health system, which requires governmental efforts to invest in more resources and infrastructures to enhance its capacity and function and be better prepared for future epidemics.
